# Survival after Left Ventricular Free Wall Rupture in an Elderly Woman with Acute Myocardial Infarction Treated Only Medically

**DOI:** 10.1155/2012/728602

**Published:** 2012-03-26

**Authors:** Víctor Hugo Roa-Castro, Ervin Molina-Bello, Hector Valenzuela-Suárez, Tobías Rotberg-Jagode, Nilda Espinola-Zavaleta

**Affiliations:** ^1^Department of Clinical Cardiology, The American British Cowdray Medical Center IAP, Campus Observatorio, Sur 136 No. 116, Colonia Las Americas, Delegación Alvaro Obregón, 01120 Mexico City, DF, Mexico; ^2^Departamento de Ecocardiografía, Instituto Nacional de Cardiología Ignacio Chávez, Juan Badiano No. 1, Colonia Sección XVI, Delegación Tlalpan, 14080 Mexico City, DF, Mexico

## Abstract

Pseudoaneurysm of the left ventricle is rare and may occur as a result of transmural myocardial infarction. The course of rupture after acute myocardial infarction varies from a catastrophic event, with an acute tear leading to immediate death (acute rupture), or slow and incomplete tear leading to a late rupture (subacute rupture). Incomplete rupture may occur when the thrombus and haematoma together with the pericardium seal the rupture of the left ventricle and may develop into a pseudoaneurysm. Early diagnosis and treatment is essential in this condition. Two-dimensional color Doppler echocardiography is the first-choice method for most patients with suspected left ventricular pseudoaneurysm (LVP) and suggests left ventricular rupture in 85% to 90% of patients. We report the case of an 87-year-old woman presenting with symptoms and findings of myocardial infarction and left ventricular free wall rupture with a pseudoaneurysm formation diagnosed by echocardiography and confirmed on CT, MRI, and NM. She received only intense medical treatment, because she refused surgery with a favorable outcome. After 24-month followup, she is in NYHA functional class II. The survival of this patient is due to the contained pseudoaneurysm by dense pericardial adhesions, related to her previous coronary bypass surgery.

## 1. Background

Left ventricular free wall rupture (LVFWR) in myocardial infarction (MI) is often fatal, and only a few patients may undergo operation. The cardiac rupture may be clinically undetected and lead to pseudoaneurysm [1–3]. Left ventricular pseudoaneurysm (LVP) is formed when cardiac rupture is contained by adherent pericardium or scar tissue [4]. Two-dimensional echo is the first-choice method for patients with suspected LVP and suggests left ventricular rupture in 85% to 90% of patients [5]. The potential use of 3D echo in assessing the location and complex geometry of ventricular rupture site has been demonstrated [6]. The main aim of this case is to describe the long survival of a woman in the ninth decade of life with acute LVFWR and LVP formation after MI.

## 2. Case Report

**Figure 1:**
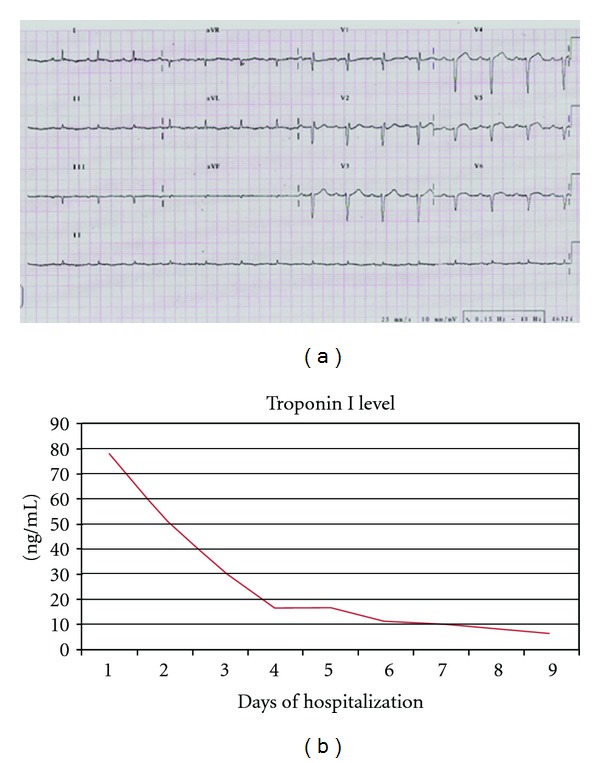
(a) Surface electrocardiogram in sinus rhythm with QS waves in III and aVF, QS waves in V1–V6 elevation of ST segment of <0.1 mv in V3 to V6, atrioventricular first degree block and asymmetric inversion of T wave in I and aVL. (b) Troponin I level evolution during hospitalization.

**Figure 2:**
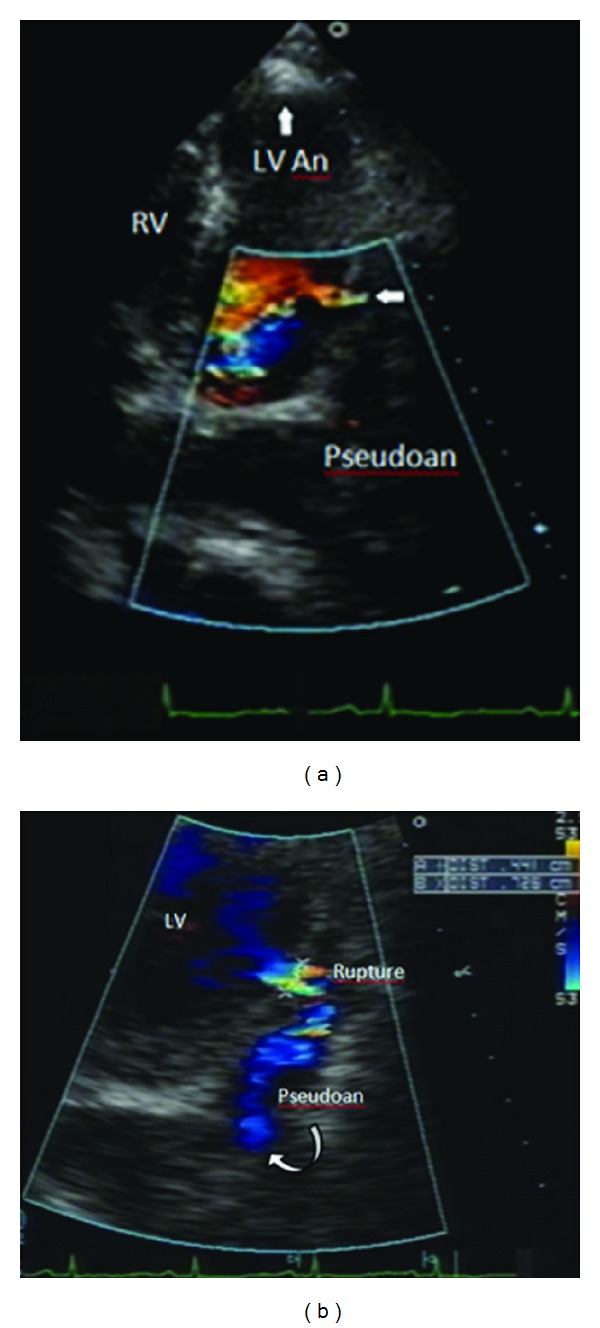
(a) Transthoracic two-dimensional and color Doppler echocardiogram in four-chamber view, showing the left ventricular apical aneurysm (LVAn), the site of rupture (arrow) and the pseudonaeurysm formation (curved arrow). (b) Zoom in lateral wall rupture with color Doppler.

**Figure 3:**
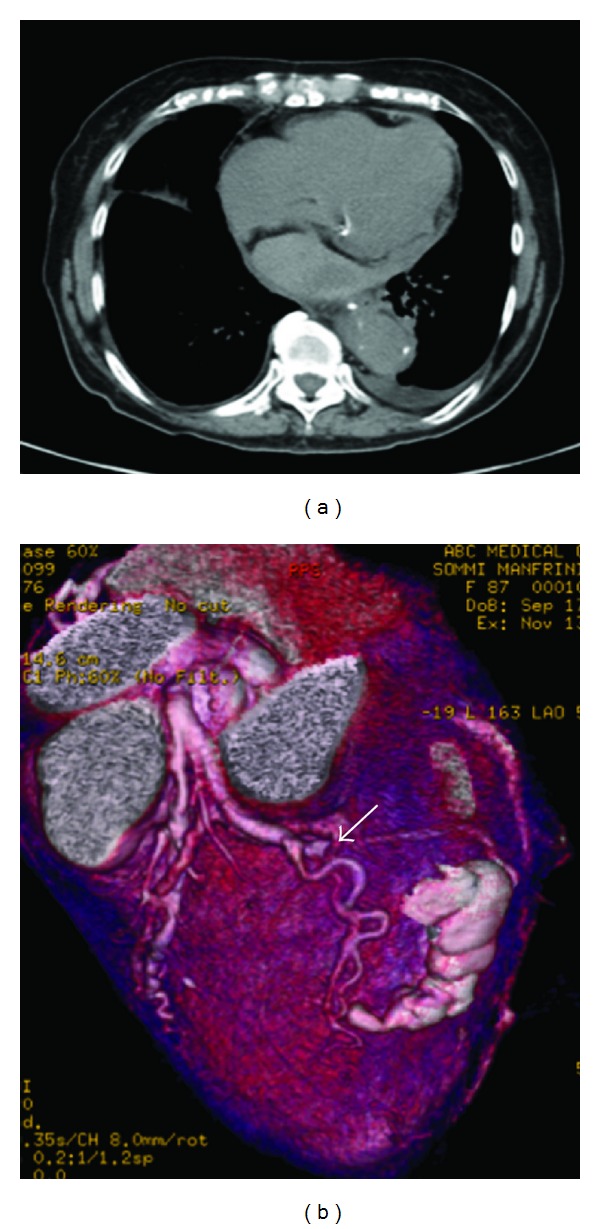
In (a) an important thickening of the pericardium containing the hemopericardium is seen and in (b) the cardiac hybrid CTA image shows a significative obstruction of circumflex artery in its middle segment (white arrow).

**Figure 4:**
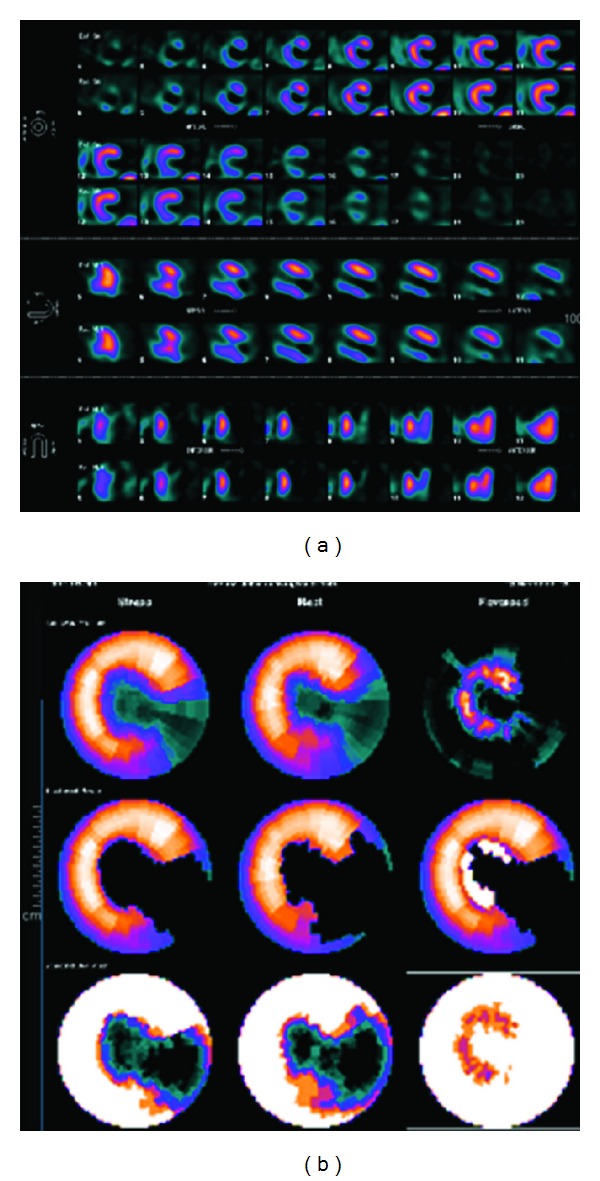
(a) The myocardial perfusion imaging with TC-99 M Sestamibi SPECT at rest and stress with dipyridamole demonstrated left ventricular lateral wall and apical transmural myocardial infarction without peri-infarction myocardial ischemia. (b) Bull's eye plots were created by automated quantitative analysis software, representing the stress and rest data.

**Figure 5:**
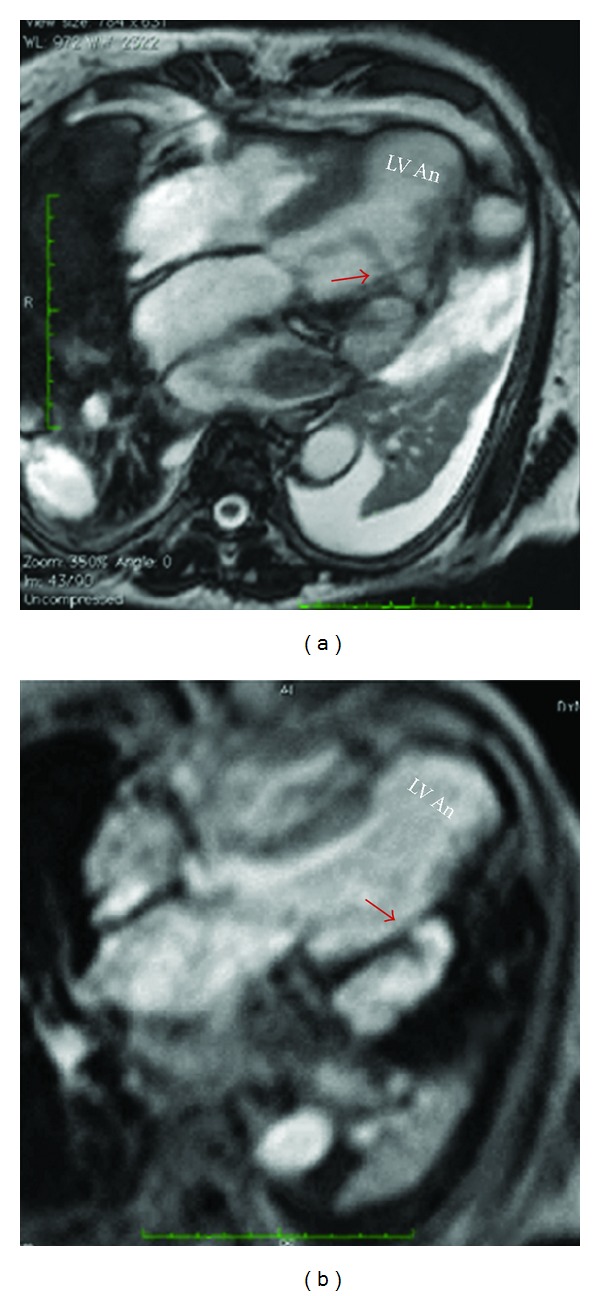
(a) Four-chamber view cardiac MR cine SSFP shows an apical aneurysm (LV An) and a lateral left ventricular pseudoaneurysm (red arrows). Apical transmural hyperenhancement is also seen in (b).

**Figure 6:**
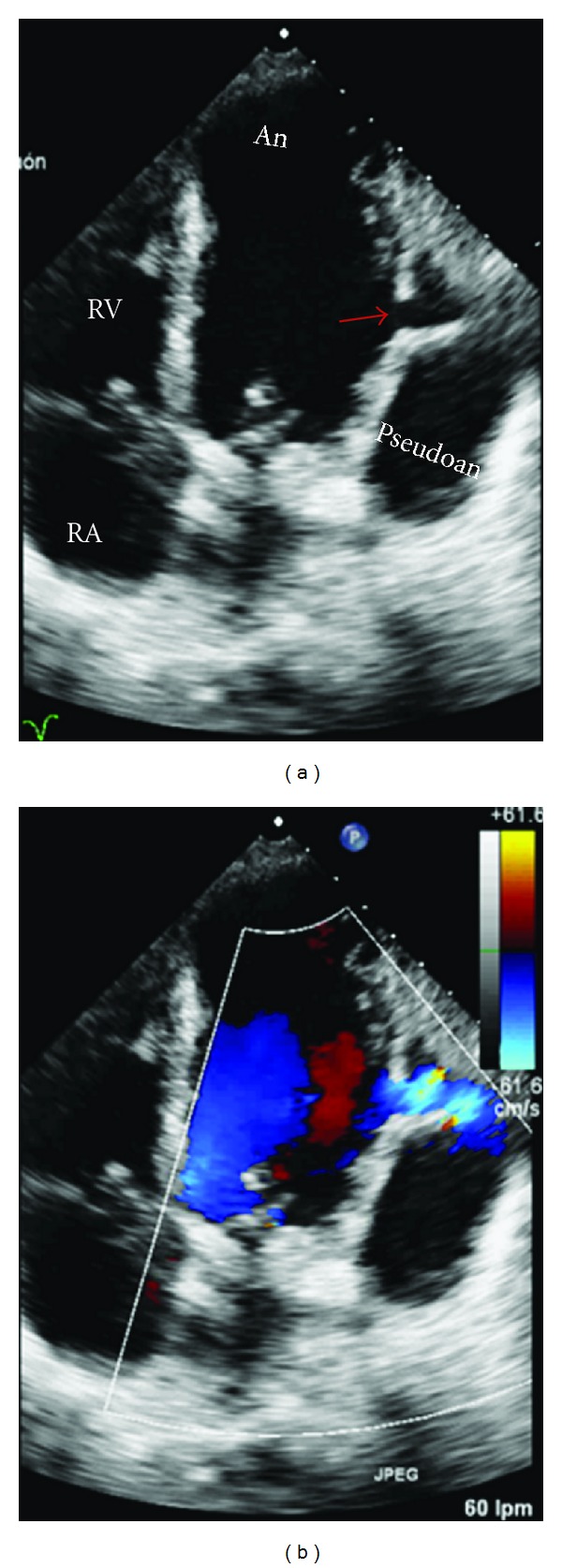
Two-dimensional (a), and color Doppler four-chamber view (b) images showing an apical aneurysm (LVAn) and lateral pseudoaneurysm formation (red arrow) with shunt from the left ventricle to the pericardial space. RV-right ventricle, RA-right atrium, Pseudoan-pseudoaneurysm.

**Figure 7:**
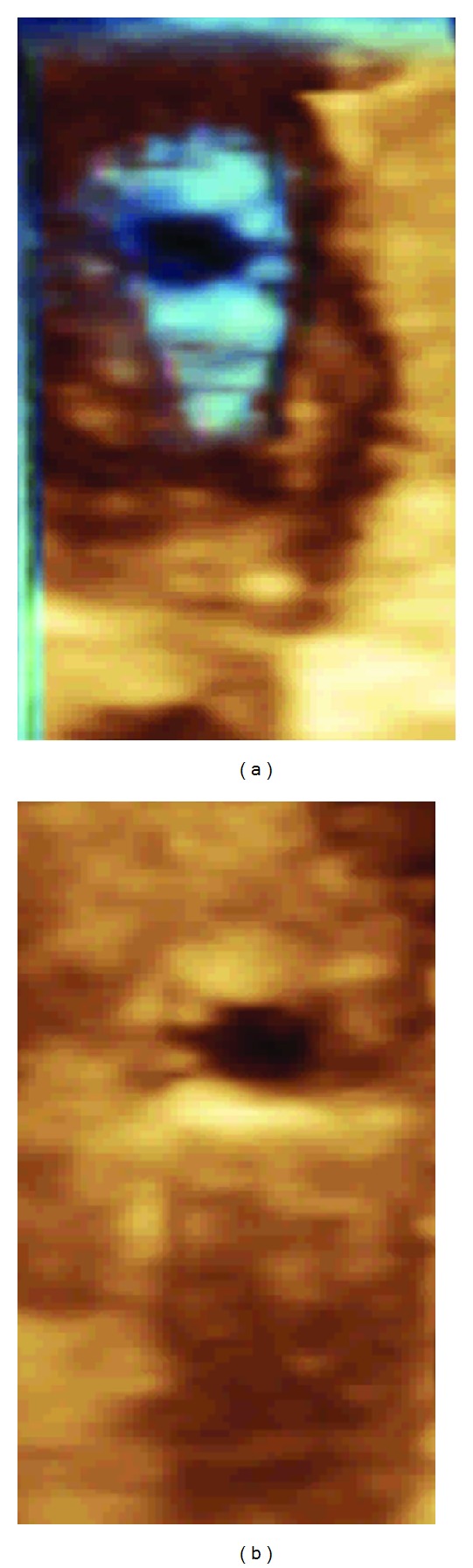
Real time 3D internal (a) and external (b) echocardiographic views of the left ventricular free wall rupture, which has a circular geometry.

An 87-year-old woman with history of hypothyroidism, systemic arterial hypertension, anterior MI with an LV apical aneurysm, and coronary artery bypass graft to the left anterior descending in 1997 presented to the emergency room with an epigastric discomfort that had begun 24 hours earlier and a diagnosis of acute MI was made. At admission she was hemodynamically stable. Vital signs included a BP of 130/70 mmHg, HR 70 beats/min, RR of 16, temperature of 36.5°C, and oxygen saturation of 92% on room air. On heart auscultation, an S3 was heard. Electrocardiogram showed q wave in III, aVF, asymmetric wave inversion in leads I and aVL and elevation of ST segment of <0.1 mv in V3 to V6, (Figure 1(a)). Elevation of cardiac troponin I level to 78 ng/mL was found normal: <0.04 ng/mL (Figure 1(b)). A 2D echo (SONOS 5500, Philips Medical Systems, Bothell, Washington, USA) performed at the bedside in the emergency room showed the site of LVFWR, the blood flow from the LV to the pericardial space and diastolic flow from the pericardial space to the LV with hemopericardium contained by echo-dense pericardial adhesions, and an LV apical aneurysm (Figure 2). A cardiac CT was done to assess the coronary anatomy and the pericardium, which showed a total occlusion of circumflex artery in its middle segment (Figure 3) and an important thickening of the pericardium at the LV lateral wall. An intense medical treatment with ACE inhibitors, diuretics, digitalis glycosides, statins, nitroglycerin, aspirin, and clopidogrel was administered. At 5th day of hospitalization the patient presented with cardiogenic shock, which had a good response to a 24-hours infusion of levosimendan (0.2 mcg/Kg/min). The myocardial perfusion imaging with TC-99 M Sestamibi SPECT at rest and stress with dipyridamole demonstrated LV lateral wall and apical transmural MI without peri-infarction myocardial ischemia (Figure 4). The serial echo studies and the MRI did not show any progression of the LVP (Figure 5). At 12th day of hospitalization she was discharged in NYHA functional class II, with prescription of ACE inhibitors, beta blocker, diuretics, statins, and aspirin. During a follow-up period of 24 months the patient continues to be in NYHA functional class II, and the 2D and 3D echo studies performed with a IE33 Philips Medical Systems confirmed a circular lateral wall rupture (Figures 6 and 7, and see supplementary material, movie clip Figures 6 and 7 available online at doi: 10.1155/2012/728602). 

## 3. Discussion

Left ventricular free wall rupture occurs in up to 10% of the in-hospital deaths following acute MI (usually between 3 to 6 days) and the survival is associated with emergency operation [7]. In a large review of cases, the distribution of free-wall rupture location was inferolateral (posterior) wall segment (43%), lateral wall (28%), then apical wall (24%) followed by other segments at equal frequency [5]. In our case the location of LVFWR was lateral wall.

Transthoracic echo with Doppler is the noninvasive method of choice for patients with suspected LVP. The use of real-time 3D echo in assessing the complex geometry of ventricular rupture site has been demonstrated [6, 8]. The current case shows the potential of 2D and real-time 3D echo for the assessment of the location of LV rupture, orifice geometry, and complex intracardiac flow. The 12-months follow-up real-time 3D echo study clearly demonstrated the site and circular geometry of the LVFWR.

The MRI showed the necrotic zones around the site of rupture, the MN study had not detect myocardial viability and the cardiac CT was very useful in the assessment of the coronary artery lesions and in the characterization of the pericardium, especially at the level of LVP.

Based on current literature, pseudoaneurysms are a surgical emergency and even though the scar tissue may have closed the rupture, it is just one case out of the large majority that needs to be treated aggressively. However, the postoperative mortality after surgical repair of a pseudoaneurysm ranges from 13% to 29% and may be even higher in many hospitals due to a lack of experience [9].

Taking into consideration the relatively high risk of stroke and chronic anticoagulant treatment in our elderly patient and her disagreement with the surgical repair a conservative management was administered and after 24 months of followup she is in NYHA functional class II.

## 4. Conclusions

Our patient illustrates the usefulness of noninvasive imaging modalities for the diagnosis and followup of an LV pseudoaneurysm which is equivalent to the invasive contrast technique and we believe that the long survival of this oldest patient in the literature [10, 11] is due to the contained pseudoaneurysm by dense pericardial adhesions, related to her previous coronary bypass surgery. The optimum medical therapy is the only alternative in those high-risk patients who refuse surgical operation as occurred in our patient.

## Supplementary Material

Movie Clip Figure 6: Transthoracic two-dimensional and color Doppler flow video clip in four chamber view shows an apical aneurysm and lateral pseudoaneurysm formation with shunt from the left ventricle to the pericardial space.
Movie Clip Figure 7: Real time 3D video clip of the left ventricular free wall rupture with a circular shape.

## Abbreviations

LVFWR: Left ventricular free wall rupture
LVP: Left ventricular pseudoaneurysm
CT: Computed tomography
MRI: Magnetic resonance imaging
NM: Nuclear medicine
NYHA: New York Heart Association
MI: Myocardial infarction
LV: Left ventricle
ACE: Angiotensin converting enzyme
SPECT: Single-photon emission computed tomography
BP: Blood pressure
HR: Heart rate
RR: Respiratory rate
S3: Third sound
2D: Two dimensional
3D: Three dimensional
LVAn: Left ventricular apical aneurysm
RV: Right ventricle
RA: Right atrium
Pseudoan: Pseudoaneurysm.
